# Reactive Oxygen Species in Anticancer Immunity: A Double-Edged Sword

**DOI:** 10.3389/fbioe.2021.784612

**Published:** 2021-11-17

**Authors:** Jie Wang, Ning Liu, Hongfei Jiang, Qian Li, Dongming Xing

**Affiliations:** ^1^ The Affiliated Hospital of Qingdao University, Qingdao University, Qingdao, China; ^2^ Qingdao Cancer Institute, Qingdao University, Qingdao, China; ^3^ School of Life Sciences, Tsinghua University, Beijing, China

**Keywords:** ROS, immunotherapy, macrophage polarization, T cell activation, immunogenic cell death

## Abstract

Reactive oxygen species (ROS) are critical mediators in many physiological processes including innate and adaptive immunity, making the modulation of ROS level a powerful strategy to augment anticancer immunity. However, current evidences suggest the necessity of a deeper understanding of their multiple roles, which may vary with their concentration, location and the immune microenvironment they are in. Here, we have reviewed the reported effects of ROS on macrophage polarization, immune checkpoint blocking (ICB) therapy, T cell activation and expansion, as well as the induction of immunogenic cell death. A majority of reports are indicating detrimental effects of ROS, but it is unadvisable to simply scavenge them because of their pleiotropic effects in most occasions (except in T cell activation and expansion where ROS are generally undesirable). Therefore, clinical success will need a clearer illustration of their multi-faced functions, as well as more advanced technologies to tune ROS level with high spatiotemporal control and species-specificity. With such progresses, the efficacy of current immunotherapies will be greatly improved by combining with ROS-targeted therapies.

## Introduction

Reactive oxygen species (ROS) are a class of highly reactive oxygen-derived chemicals, including hydroxyl radical (·OH), singlet oxygen (^1^O_2_), superoxide anion (O_2_^·−^), and peroxides. A group of biological reactions, with the oxidative metabolisms within mitochondria being a major source, can generate ROS in human body. Despite being byproducts in many occasions, ROS at suitable concentrations and locations are vital messengers in cellular signaling and can trigger important biosynthetic processes such as the crosslinking of extracellular matrix (Schieber and Chandel, 2014; Zhou et al., 2020). On the other hand, given the high reactivity of ROS that can be harmful to protein, DNA, and lipids, an antioxidant system has been built to maintain the homeostasis of ROS generation and elimination (Yu et al., 2020). Under pathological conditions, the delicate balance will be disturbed and usually lead to ROS accumulation and oxidative stress (Aggarwal et al., 2019). In oncology, evidence has linked the increased ROS level with cancer initiation, progression, angiogenesis, and metastasis (Moldogazieva et al., 2018), making ROS elimination a promising strategy for controlling the disease (Zheng et al., 2021). Paradoxically, ROS can also be beneficial for tumor suppression. For example, the expression of many tumor suppressor genes (e.g., *p53*) is controlled by ROS (Liu et al., 2008; Perillo et al., 2020); many drugs including chemotherapeutic and radiotherapeutic agents kill cancer cells by elevating ROS level; (Ji et al., 2021; Perillo et al., 2020) etc.

Cancer immunotherapy strengthens one’s own immune system to recognize and attack tumor cells. The last decade has witnessed the rapid development of immunotherapy with tens of different therapeutics at various treatment modalities been approved by regulatory administrations for clinical use (Smyth et al., 2016; Waldman et al., 2020; Yang et al., 2021). Interestingly, ROS play multiple roles in immunity and can be explored as potent targets to augment the magnitude and specificity of antitumor response (Kotsafti et al., 2020). A large number of studies have reported the benefits of ROS in anticancer immunity; however, the paradox still exists. The often-encountered immunosuppression, such as the attenuated T cell activation and activity (Qu et al., 2013), raises a necessity for researchers to build a clearer illustration about which role will ROS play under a given condition. This review summarizes recent studies reporting ROS-mediated enhancement or attenuation of antitumor immunity, with an expectation of providing basic rationales for improved immunotherapy.

## Interlacing Roles of ROS in Immunotherapy

Among the multiple fields ROS are functioning, the following four are of particular significance.

### Macrophage Polarization

Macrophages play critical roles in tissue homeostasis by regulating tissue development, mediating inflammatory responses and clearing pathogens and cell debris (DeNardo and Ruffell, 2019; Zheng et al., 2021). They are inducible in function, with the classically activated M1 type exerting pro-inflammatory and antitumor activities while the M2 type functioning basically the opposite (Mills et al., 2016). Local ROS concentration has an obvious influence on the polarization of macrophages, and based on current evidences ROS may induce pro-inflammatory macrophages more dominantly than doing the opposite. They can activate nuclear factor κB (NFκB) and p38 mitogen-activated protein kinase (MAPK) signaling pathways and promote the expression of M1-associated pro-inflammatory cytokines and chemokines (Rendra, et al., 2019). This mechanism is widely accepted in innate immunity and has also been reported to augment the antitumor immunity. For example, iron overload, which rapidly induced ROS production, polarized macrophages to pro-inflammatory phenotype, enhanced the activity of p300/CBP acetyltransferase and improved *p53* acetylation (Zhou et al., 2018). However, other studies indicated the M2-promoting function of ROS. Typical studies involving diverse M2-promiting mechanisms have been summarized in Table 1.

**TABLE 1:** Representative studies reporting the ROS-promoted M2 polarization of macrophages.

Model	Tested markers	ROS modulation	Mechanisms of M2 polarization	References
Mouse bone marrow-derived macrophages	M1: CD86, TNF-*α*, IL-12; M2: IL-10, CCL17/18/24	O_2_^•−^ increment by NOX; elimination by BHA	ROS induce late-phase activation of ERK signaling	Zhang, et al. (2013)
Mouse RAW 264.7 macrophages	M1: CD11b; M2: CD206, Arg-1	mtROS; reduction by antioxidant	Antioxidant reduce M2 type *via* ROS/ERK and mTOR pathway	Shan, et al. (2017)
Primary human macrophages	M1: TNF-*α*, IL12b; M2: CD163, CD206	Increased *via* H_2_O_2_ addition; reduced using MnTe	Presumably induce Stat3 activation for M2 polarization	Griess, et al. (2020)
Monocytes in human peripheral blood mononuclear cells	M1: not tested; M2: CD163, CD206	Increment *via* CAF stimulation; reduction by BHA	Not directly tested	Zhang, et al. (2017)
Mouse bone marrow-derived macrophages	M1: IL-6; M2: Arg-1, Mrc1, IL-10, Ym2, Fizz1	mtROS increment *via* GMFG knockdown; reduction by antioxidant	Increased mtROS presumably alters iron metabolism-related protein expression	Aerbajinai, et al. (2019)
Murine peritoneal macrophage	M1: IL-6, TNF-*α*, IL-1*β*; M2: Arg-1, Ym1, Fizz1-Relm-*α*	MCPIP-stimulated ROS production	ROS induced ER stress and autophagy to increase M2 markers	Kapoor, et al. (2015)

NOX: NADPH, oxidase; Arg-1: arginase-1; mtROS: mitochondria ROS; mTOR: mammalian target of rapamycin; MnTe: MnTE-2-PyP^5+^; Stat3: signal transducer and activator of transcription 3; CAF: cancer-associated fibroblasts; GMFG: glia maturation factor-*λ*; MCPIP: MCP-1-induced protein.

Therefore, ROS can induce the differentiation of macrophages to both M1 and M2 types, raising uncertainty for the direction of ROS modulation (Rendra et al., 2019; Zhou et al., 2020). What further complicates the situation is that factors inducing M1 polarization may not provide benefits for cancer suppression. For example, it was shown that black raspberries, which served as an antioxidant, reduced the incidence of esophageal cancer by suppressing oxidative stress and NFκB/MAPK signaling (Shi, et al., 2017). Given the presence of pathways that lead to contrary results, it can be envisioned that ROS may simultaneously exert opposite influences on macrophages, and the ultimate impact may depend on ROS concentration (including the relative concentration of different species), location and their interaction with therapeutic agents. It is noteworthy that besides M1/M2 polarization, ROS influence macrophages in many other aspects. Roux et al. showed that ROS mediated the immunosuppression effect of macrophages by up-regulating the expression of programmed death ligand-1 (PD-L1) (Roux et al., 2019). When treated with ROS inducers such as paclitaxel, PD-L1 expression was up-regulated on the surface of tumor-associated (TAMs) in a mouse model of triple negative breast cancer, *via* the activation of NFκB signaling. Note that both M1 and M2 signatures positively correlated with the expression of PD-L1.

### Efficacy of Immune Checkpoint Blockades

Using monoclonal antibodies to block the immune checkpoint-mediated immune escape has been one of the most promising strategy for tumor control (Havel et al., 2019). The immune microenvironment exerts a great influence on the treatment efficacy, while ROS serve as critical mediators. A large number of studies have shown that ROS generation would lead to augmented expression of the programmed death-ligand 1 (PD-L1) on cancer cells. A straight forward mechanism is that ROS elevation leads to the upregulation or stabilization of multiple transcription factors such as NFκB and HIF-1*α*, while NFκB initiates PD-L1 expression by binding to the PD-L1 promoter (Guo et al., 2019), and HIF-1*α* directly binds to a transcriptionally active hypoxia-response element in the PD-L1 proximal promoter (Noman et al., 2014). Note that hypoxia-induced HIF-1*α* activation can either elevate ROS level *via* NOX or reduce ROS by inhibiting the tricarboxylic acid cycle (Chen et al., 2018). There are also studies reporting enhanced ROS generation with reduced PD-L1 expression or vice versa, as summarized in a review article (Bailly, 2020). Nevertheless, in most occasions the PD-L1 expression is positively correlated with ROS level, as demonstrated by using a large variety of ROS-modulating small molecules and human oncoviruses (Montani, et al., 2018). Meanwhile, the role macrophage plays in the connection between ROS and PD-L1 expression is worth noting. ROS is a critical mediator of macrophage polarization, while PD-L1 high expression has been found to be correlated with M2-polarization of macrophages (Zhu, et al., 2020). Therefore, skewing the M1/M2 balance of macrophages may be a potential route by which ROS modulate PD-L1 expression.

ROS also have an impact on programmed death-1 (PD-1) blocking therapy at least due to the ligand/receptor relationship of PD-L1/PD-1. Chamoto et al. reported that tumor-reactive T cells boosted by PD-L1 blockade possessed activated mitochondria with augmented ROS production, and improving ROS generation using ROS precursors or mitochondrial uncouplers synergized the antitumor activity of PD-1 blockade by expansion of effector/memory cytotoxic T cells in draining lymph nodes (Chamoto et al., 2017). A more recent study reported similar results, that in a “bilateral tumor model”, ROS increment in CD8^+^ T cells was observed only in tumors that were responsive to PD-1 blockade therapy (Kumar et al., 2020). Therefore, ROS level in tumoral and lymphatic cells might be a potential indicator of the responsiveness to PD-1/PD-L1 blockade therapy, especially considering that the expression of immune checkpoints has been accepted as a tumor-intrinsic sign of the vulnerability of tumors to ICB therapy (Zappasodi et al., 2018).

Cytotoxic T lymphocyte-associated antigen-4 (CTLA-4) is a vital regulator of T cell function. Studies directly exploring the effect of ROS on CTLA-4 therapy are rare to the best of our knowledge, but ROS have an obvious influence on the development of T regulatory cells (Tregs), on which CTLA-4 is constitutively expressed (Walker 2013). The effects are still multi-faced. For example, macrophage-generated ROS were functional for the induction of Tregs (Kraaij et al., 2010), while neutrophil cytosolic factor 1-deficient mice with a lower level of ROS also carried Tregs more reactive than those from wild mice (Kim et al., 2014). Obviously, the expansion of CTLA-4 blocking therapy warrants further studies on the effects of ROS.

### T Cell Activation and Expansion

Activation of T cells, as the pivotal step in cellular immunity, relies on the binding with main histocompatibility complex, the stimulation by co-stimulatory molecules on antigen-presenting cells, and a suitable biochemical environment that allows these processes to happen. ROS have a great influence on T cell activation. An example is that the local number and phenotype of macrophages, which often function as antigen presenting cells, can be re-/programmed by ROS as described above. Another aspect is the ROS-tuned expression of immune checkpoint molecules on immune cells and cancer cells. More directly, ROS can create an oxidative environment to inactivate T cells. It was reported that the redox level on cell surface physically determines the reactivity of T cells (Sahaf et al., 2003). Researchers found that mice were more susceptible to develop severe arthritis if ROS production was diminished, and then revealed that lower ROS level would increase the number of reduced thiol groups on T cell membrane surface and make T cells more prone to be activated (Gelderman et al., 2006). This is a necessary mechanism to prevent the over-activation of T cells in inflammatory sites, while for cancer treatment this is commonly undesirable (Moro-Garcia et al., 2018; Yin et al., 2021). Artificially increasing cell surface thiol by adding antioxidants (e.g., glutathione, GSH) or reducing ROS generation has been employed to increase the sensitivity of T cells to stimulatory signals (Kesarwani, et al., 2013).

Efficient expansion of tumor-specific T cells upon activation is necessary in cell therapy, and the failure to do so has been a major limitation for adoptive cell therapy to achieve broader application. It was shown that the persistence of effector CD8^+^ T cells and CD62L^hi^ central memory T cells were obviously longer if the cytosolic GSH and surface thiol were higher (Kesarwani et al., 2015), while GSH depletion prevented T cell proliferation despite the stimulation using antigens (Mak et al., 2017). Pretreatment with antioxidant N-acetyl cysteine (NAC) during *ex vivo* T cell expansion process significantly improved the persistence of adoptively transferred cells and led to more effective tumor control in a mouse model of melanoma (Scheffel et al., 2016). The underlying mechanism was revealed to be reduction in DNA damage by reducing ROS and the resultant reduced activation-induced cell death (an immunosuppressive process known to be induced by repeated stimulation of T cell receptor) of T cells in the presence of NAC (Scheffel et al., 2016). All these evidence suggests the necessity of adding antioxidants to the culture media of therapeutic T cell survival and expansion. Note that both GSH and NAC contain thiol groups as potent reducing moiety to scavenge electrons from highly reactive molecules, e.g., to consume ROS.

### Immunogenic Cell Death

Cancer develops with mutations, resulting in the emergence of abundant neoepitopes (sequence-altered nucleic acids and proteins) that are foreign to host’s immune system. Immune responses induced by a specific neoepitope may fail to damage tumor cells that do not contain this neoepitope, while immunogenic cell death (ICD), which is featured by the release of tumor-associated antigens and danger-associated molecular patterns, will provide a full spectrum of neoepitopes to eliminate immune escape caused by tumor heterogeneity. ROS generally have an inducing effect to ICD occurrence. The induction of endoplasmic reticulum (ER) stress, surface exposure of calreticulin, and secretion of adenosine triphosphate (ATP), high-mobility group box 1 (HMGB1) and heat shock protein 70 (HSP70) are requisites for ICD (Bugaut et al., 2013; Van Loenhout et al., 2020), while many of these processes can be triggered by ROS. An example is that bleomycin (an anticancer drug relying on its ability to generate ROS) induced ER stress and autophagy, which then led to calreticulin exposure and release of HMGB1 and ATP to trigger ICD (Bugaut et al., 2013). Actually, many chemotherapeutic small molecules known to kill cancer cells *via* ROS generation are undergoing clinical trials as ICD inducers beyond chemo drugs, such as doxorubicin, bortezomib, and epirubicin (Vanmeerbeek et al., 2020). Other kinds of agents are also under exploration. For example, a fluorinated mitochondria-disrupting helical polypeptides, which could destabilize mitochondrial outer membrane, was developed to over-produce intracellular ROS (iROS), induce ICD and enhance PD-L1 blockade therapy (Jeong et al., 2021).

Particularly, a number of treatment modalities have intrinsic capability to induce ICD by producing ROS or other critical stimulators. 1) Photodynamic therapy (PDT), which kills cancer cells by generating abundant ROS with the assistance of photosensitizers and light irradiation, can induce ICD and antitumor immunity (Panzarini et al., 2013). Cellular internalization of photosensitizers causes high iROS level and ER stress especially when photosensitizers localizes near the ER. Using a ER-associated photosensitizer, hypericin, Garg et al. found that PDT generated obvious ER stress, and caused cancer cells to secrete ATP, display damage-associated molecular patterns on cell surface and undergo immunogenic apoptosis (Garg et al., 2012; Garg et al., 2012). The display of calreticulin was crucial by providing the motifs needed for the engulfment of PDT-treated cells by dendritic cells (Garg et al., 2012). Using other photosensitizers other than hypericin failed to induce the exposure of calreticulin on cell surface (Garg et al., 2012), suggesting the necessity of choosing suitable photosensitizers or choosing suitable drug carriers to afford enhanced affinity to ER. 2) Sonodynamic therapy is similar with PDT but employs ultrasound as the energy source (Li et al., 2021), and has been reported to elicit ICD. For instance, a nanocomposite loaded with chlorin e6 (as a sonosensitizer) induced ICD *via* receptor-interacting protein kinase 3–dependent cell necroptosis (Um et al., 2020). 3) Radiotherapy produces ROS *via* radiolysis and induce ICD, although the break of double-strand DNA was previously considered as the primary mechanism of tumor suppression in radiotherapy. Actually, ICD-mediated antitumor immunity has been recognized as the origin of abscopal effect in radiotherapy.

**FIGURE 1:**
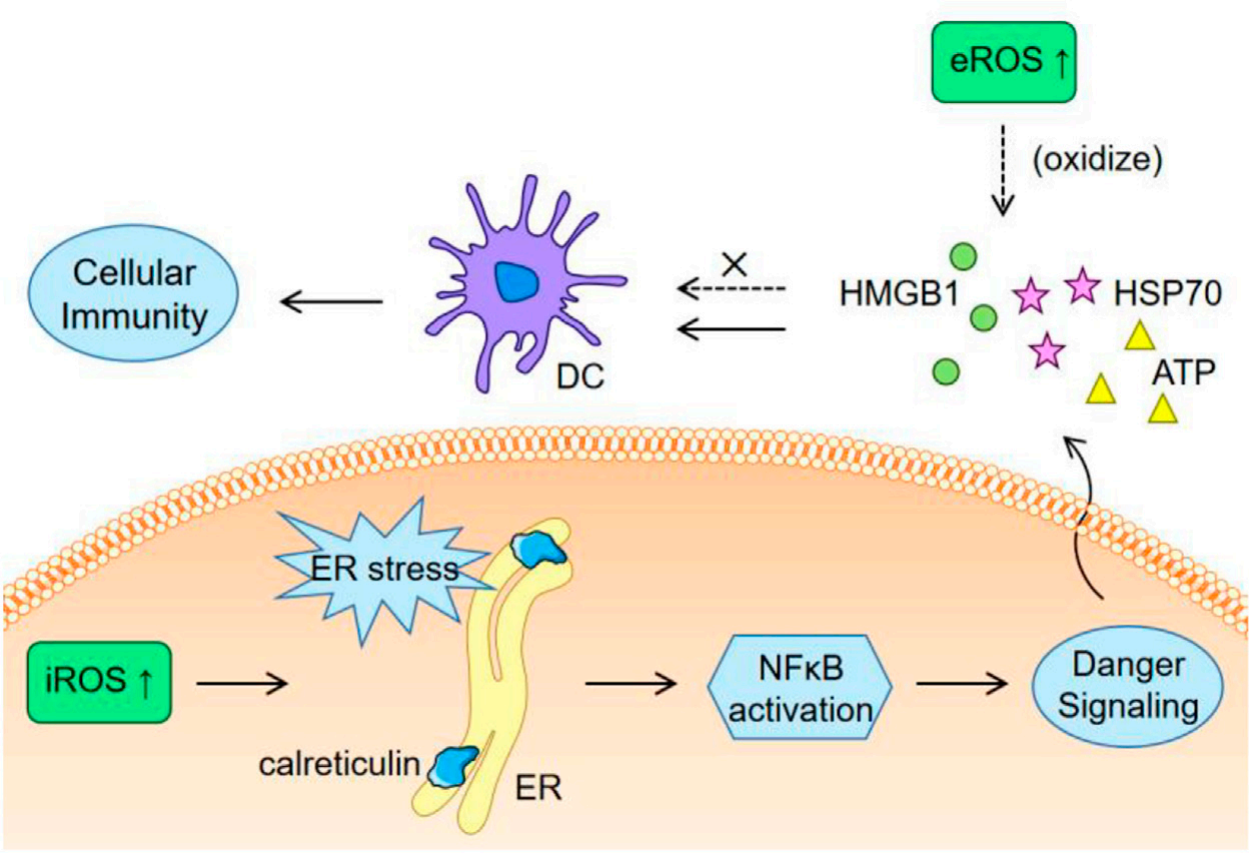
Schematic illustration of the different effects of iROS and eROS on ICD.

There are also studies reporting obvious inhibition of ICD-induced immune response by elevated ROS. Kazama et al*.* reported that ROS would neutralize the stimulatory capacity of dying cells (Kazama et al., 2008). The results showed that caspase activation against mitochondria promoted immune tolerance of apoptotic cells by generating ROS to oxidize the HMGB1 (Kazama et al., 2008). HMGB1 potently act on dendritic cells to stimulate immunity (Dumitriu et al., 2005), so its inactivation promotes immune tolerance. Using a ROS scavenger to consume extracellular ROS (eROS) enhanced the stimulatory effect of dying cells by avoiding the oxidation of HMGB1 (Deng et al., 2020). Therefore, as depicted in Figure 1, there might be a need to induce ER stress *via* iROS and simultaneously eliminate eROS to avoid the oxidization of the exposed calreticulin and the released stimulatory molecules.

## Conclusions and Perspectives

ROS are continuously generated in a large variety of biochemical reactions. Although a majority of studies are linking them to disease states such as insulin resistance, inflammation, and cancer, ROS play important roles in immune responses. This warrants a very clear understanding of the multi-faced but tunable roles of ROS. There may be more studies reporting detrimental effects of ROS on antitumor immunity than those indicating beneficial effects, since they can drive macrophages to polarize to immunosuppressive types, promote the expression of PD-L1, attenuate the efficacy of ICB therapy, deactivate T cells and inhibit the occurrence of ICD. However, it is not wise to simply scavenge ROS because they have pleiotropic effects in most cases, and also because the detrimental/beneficial switch can be easily shifted by modulating ROS concentration, location, species, and the scenarios they are in. For example, ROS can increase the expression of PD-L1, but it is unachievable to eliminate PD-L1 by scavenging ROS and doing so will greatly attenuate the immuno-stimulatory effects of ROS and cause redox imbalance-related adverse effects. Therefore, clinical application of directly tuning ROS level still has a long way to go.

Meanwhile, most of the reported works have studied ROS as a whole without distinguishing their species, possibly due to the limited specificity of detection probes (e.g., 2',7'-dichlorodihydrofluorescein) to ROS species (which include ·OH, O_2_^·−^ and H_2_O_2_). Free radicals (·OH and O_2_^·−^) can readily trigger chain reactions and produce various carbon-centered radicals, while H_2_O_2_ are relatively long-lived and inactive compared with free radicals and commonly exert oxidative capability with the assistance of metal ions such as iron and copper. Such a chemical basis provides a good reason to consider that different species will cause varied magnitude of oxidative stress and mediate distinct signaling pathways (Collin, 2019). The location of the studied ROS is another parameter being important but easily ignored. For example, ROS-producing nanomaterials are widely employed to treat cancer, while the main location (e.g., intracellular or extracellular; intra-lysosomal or intracytoplasmic) is hard to determine since the cellular internalization rate and lysosomal escape efficiency of nanomaterials are difficult to quantify. In this context, choosing biomaterials as ROS inducers with well-defined pharmacokinetics will help. With the building of such theoretical rationales and technical capabilities, ROS-targeted therapy will eventually synergize with current immunotherapies.
